# Validity of prognostic scoring systems used in critically ill patients with acute on chronic liver failure

**DOI:** 10.1186/2197-425X-3-S1-A835

**Published:** 2015-10-01

**Authors:** EA Keough Delgado, P García Olivares, M Dalorzo Gonzalez, C Ramirez Gonzalez, C Caciano Reategui, M Díaz Cámara, JC Barrios Torres, C Delgado Albañil, JE Guerrero Sanz

**Affiliations:** HGU Gregorio Marañón, Intensive Care Unit, Madrid, Spain

## Introduction

Risk prediction of mortality through the use of scoring systems in the intensive care unit (ICU) is important in cirrhotic patients with acute decompensation. This is especially relevant due to the poor prognosis associated with extrahepatic organ failure and severity of hepatic dysfunction.

## Objectives

The purpose of this study was to assess the predictive prognostic value of the different severity scoring systems used in the ICU and those more specific to cirrhotic patients.

## Methods

Descriptive study of a cohort of patients admitted to ICU with a diagnosis of acutely decompensated liver cirrhosis between the years 2010-2013. Data collected included demographics, presence of comorbidities, severity of illness (APACHE II, SOFA, MELD and CLIF-SOFA), etiology as well as cirrhosis stage, reason for admission, length of stay and mortality in ICU.

Descriptive statistics were expressed as mean ± SD or median (interquartile range) for continuous variables and percentages for categorical data. The ability of the scoring systems to discriminate prognosis was assessed using the area under the receiver operating characteristic (AUROC) curve. Estimation of their calibration was established through the Hosmer-Lemeshow goodness of fit test. To evaluate the extent to which the scoring systems were valid for prediction of mortality the sensitivity (S), specificity (E), overall correctness of prediction, positive and negative predictive values (PPV, NPV) were all determined.

## Results

Sixty-one patients. 79% were male. Age 55 ± 10 yrs. Charlson Comorbidity Index: 5 ± 2 pts. Admission severity scores: APACHE II 22 ± 10 pts; SOFA 11 ± 4 pts; MELD 20 ± 8 pts; CLIF-SOFA 12 ± 4 pts. Etiology of cirrhosis: Alcohol 55% and HCV 20%. Cirrhosis stage: CHILD A 26%, CHILD B 39% and CHILD C 34%. Reason for ICU admission: upper gastrointestinal haemorrhage 38%, hepatic encephalopathy 33% and sepsis 23%. 89% of patients required mechanical ventilation (8 days; IQR 3-15) and 77% vasopressor therapy (3 days; IQR 2-6). Length of ICU stay 7 days (IQR 3-14) and 49.2% mortality in ICU.

We confirmed that all severity scores analysed were predictive of mortality: **APACHE II** (OR 1.05; CI 95% 1.01-1.12), **SOFA** (OR 1.62; CI 95% 1.26-2.10), **MELD** (OR 1.16; CI 95% 1.05-1.28), **CLIF-SOFA** (OR 1.58; CI 95% 1.24-2.01).

Using the AUROC curves, the SOFA score on admission was found to be the most reliable scoring system to discriminate mortality in ICU (AUROC 0.82, CI 0.70-0.94), with a good calibration ability (Chi-squared 7.09, p = 0.53) and with better performance indicators (S 77%, E 81%, PPV 79%, NPV 78%) (Table 1).

**Table 1:** Table 1

DISCRIMINATION			INDICATORS					
	AUROC	CI 95%	p	S	E	PPV	NPV	
APACHE II	0.66	0.50-0.81	12.54	0.08	60%	77%	72%	67%
SOFA	0.82	0.70-0.94	7.09	0.53	77%	81%	79%	78%
MELD	0.74	0.60-0.88	13.16	0.11	60%	71%	62%	68%
CLIFSOFA	0.80	0.68-0.92	8.57	0.38	73%	81%	79%	76%

## Conclusions

Our data show that the severity scores designed specifically for liver cirrhosis were not superior to the standard scoring systems used in critically ill patients. Of all the severity scores analysed, the greatest discriminatory power was observed with the SOFA score.

